# Genetic Analysis as a Tool to Improve the Monitoring of Stranded Cetaceans in Chile

**DOI:** 10.3390/biology12050748

**Published:** 2023-05-19

**Authors:** Sebastián Kraft, Francisca Rodríguez, Carlos Olavarría, Elie Poulin, María José Pérez-Álvarez

**Affiliations:** 1 Centro de Ciências do Mar (CCMAR), Universidade do Algarve, 8005-139 Faro, Portugal; 2 Laboratorio de Ecología Molecular, Departamento de Ciencias Ecológicas, Instituto de Ecología y Biodiversidad, Facultad de Ciencias, Universidad de Chile, Santiago 7800003, Chile; 3 Millennium Institute Biodiversity of Antarctic and Subantarctic Ecosystems (BASE), Santiago 7800003, Chile; 4 Centro de Estudios Avanzados en Zonas Áridas (CEAZA), La Serena 1720256, Chile; 5 Eutropia, Centro de Investigación, Santiago 8320238, Chile; 6 Escuela de Medicina Veterinaria, Facultad de Medicina y Ciencias de la Salud, Universidad Mayor, Santiago 8580745, Chile

**Keywords:** stranding record, government institution, species and sex identification, multidisciplinary approach, conservation, management

## Abstract

**Simple Summary:**

Cetacean strandings are regularly recorded along the coast of Chile. However, crucial information such as species and sex of the individuals involved in these events can often be difficult to assess. In this context, the use of molecular tools as a complementary method can improve a stranding database, particularly by correcting misidentifications and providing new data for unidentified samples. This new information is especially important in the case of species that are poorly known or of high conservation interest. In this study, we evaluate how molecular tools can support and complement the field work records of strandings in Chile by identifying, corroborating, or correcting the identification of the species and sex of the recorded individuals. We obtained samples through a collaboration with the government agency that is in charge of assisting with cetacean strandings and collected the relevant information. Multidisciplinary approaches like this, and inter-institutional collaborations, can improve the study of cetacean strandings and the decisions in management and conservation policies around them.

**Abstract:**

Cetacean strandings are a valuable source of information for several studies from species richness to conservation and management. During the examination of strandings, taxonomic and sex identification might be hindered for several reasons. Molecular techniques are valuable tools to obtain that missing information. This study evaluates how gene fragment amplification protocols can support the records of strandings done in the field in Chile by identifying, corroborating, or correcting the identification of the species and sex of the recorded individuals. Through a collaboration between a scientific laboratory and government institution in Chile, 63 samples were analyzed. Thirty-nine samples were successfully identified to the species level. In total, 17 species of six families were detected, including six species of conservation interest. Of the 39 samples, 29 corresponded to corroborations of field identifications. Seven corresponded to unidentified samples and three to corrected misidentifications, adding up to 28% of the identified samples. Sex was successfully identified for 58 of the 63 individuals. Twenty were corroborations, 34 were previously unidentified, and four were corrections. Applying this method improves the stranding database of Chile and provides new data for future management and conservation tasks.

## 1. Introduction

Cetaceans can strand alive or dead on coasts and other areas outside of their usual habitat [1] for several reasons. Possible influencing factors are navigational errors, sickness, coastal configuration, climate events, anthropogenic factors, among others [1]. Stranding events are regarded as valuable sources of information [2,3], as the data that are collected can be used for a wide range of studies, including species richness [4], identification of rare species [5], life-history traits [6], estimate growth curves [7], stock structure [8], and even as an early warning for human health hazards [9]. Additionally, much of what is currently known about several species comes from strandings [10,11], of which toothed whales (Ziphiidae; [12]), pilot whales (*Globicephala* spp.) [13,14,15], and false killer whales (*Pseudorca crassidens*) [1] are prime examples. Accordingly, our perception of strandings and relationship with them have changed and developed over time [1,16]. For example, the collection and organization of stranding information have improved [16], resulting in detailed and long-dating records of strandings in areas such as the United Kingdom [17], United States [10], New Zealand [18], and the Netherlands [19]. 

However, taxonomic and sex identification might be hindered during the examination of strandings because of advanced states of decomposition [20], lack of diagnostic features because of missing or concealed body parts, morphological similarity between species, or inexperience of the observer [21,22]. In these situations, complementary methodologies can be implemented to overcome these difficulties, such as molecular tools [23] as described in this study. In this process, specific gene fragments of a small sample of DNA can be amplified via the Polymerase Chain Reaction (PCR). This produces millions of copies of the target gene fragment that can be analyzed to identify the species and sex of the sample. 

Molecular techniques have been used, for example, to differentiate the two morphologically similar species of pilot whales (*Globicephala* spp.) in strandings that have occurred where their distributions overlap [22], to confirm the first record of a True’s beaked whale (*Mesoplodon mirus*) for New Zealand waters [21], and even to unveil the presence of a fin-blue whale hybrid [24], demonstrating the valuable information that this method can contribute. 

In Chile, cetacean strandings are recurrent events that have been recorded along the complete coastline but are particularly common in the southern regions of the country [9]. Since 1983, and more systematically since 2009 [9], the Chilean National Fisheries Service (SERNAPESCA, Servicio Nacional de Pesca y Acuicultura (www.sernapesca.cl, accessed on 9 April 2023)) has overseen the task of monitoring these strandings, assisted by the coastal unit of the Chilean Navy (DIRECTEMAR, Dirección de Territorio Marítimo (www.directemar.cl, accessed on 9 April 2023)). SERNAPESCA usually receives reports of marine mammal strandings, attends the locations of the events, records the number of individuals and species, collects tissue samples, and oversees the application of safety protocols. DIRECTEMAR enforces the application of protocols and polices and controls the stranding location in terms of public safety. One of the most notable events registered through this initiative was the largest mass mortality of baleen whales on record. This event occurred in Golfo de Penas, southern Chile, and involved at least 343 sei whales (*Balaenoptera borealis*) [25]. This event was important, as most of the available information on this species in the Eastern South Pacific comes from this mass mortality event [25,26]. Other notable massive stranding events have occurred along the Chilean coastline, involving species such as false killer whales (*Pseudorca crassidens*) [27] and long-finned pilot whales (*Globicephala melas*) [28], of which valuable information has also been gathered [13]. These examples show that the monitoring and recording of strandings has greatly improved over time, yet still is very dependent on factors such as site accessibility, available equipment, facilities, and presence of observers.

After the aforementioned mass mortality of sei whales, in 2015 an inter-institutional collaboration was established between SERNAPESCA and the Molecular Ecology laboratory at the University of Chile. Through this collaboration, tissue samples have been collected in the field and sent to our facilities to be analyzed using molecular tools. In this study, our main hypothesis is that the strandings data obtained on the field along the Chilean coast can be improved by applying molecular tools, such as the use of genetic techniques to identify species and sex.

Therefore, our goal in the present study is to evaluate how this methodology can support and complement the field work records of strandings by identifying, corroborating, or correcting the identification of the species and sex of the sampled individuals. 

## 2. Materials and Methods

### 2.1. Collection of Samples

**Figure 1:**
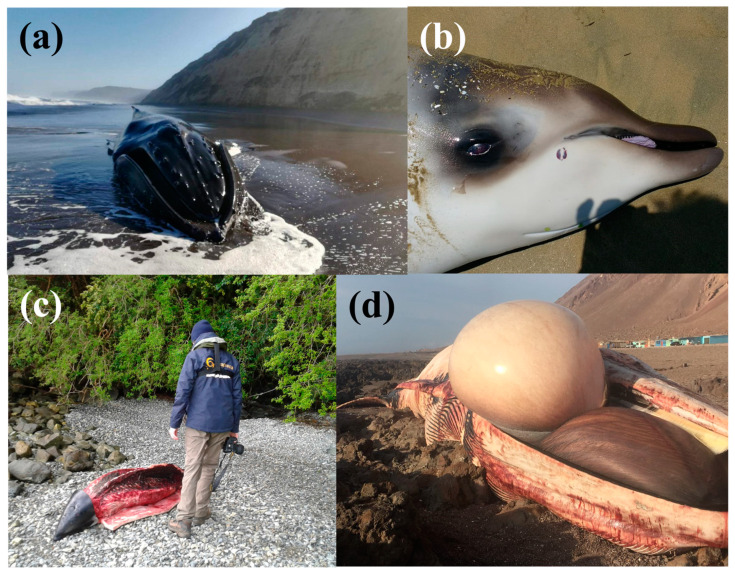
Examples of stranded cetaceans from which samples were collected, with location and author of photograph in parenthesis: (**a**) *Megaptera novaeangliae* (Santo Domingo, central Chile; Eduardo Vega); (**b**) *Mesoplodon grayii* (Coquimbo, northern Chile; Gerardo Cerda); (**c**) *Lagenorhynchus obscurus* (Bahía Acantilada, southern Chile; Natalia Toledo); (**d**) *Balaenoptera physalus* (Tocopilla, northern Chile; Sebastián Figueroa).

Tissue samples (skin) from stranded carcasses were collected by SERNAPESCA personnel, occasionally assisted by other researchers (Figure 1). For each sample, an associated data sheet was filled in the field by SERNAPESCA. The information of each skin sample used in this paper was extracted from these documents. Tissue samples were stored in 70–90% ethanol and sent to the Molecular Ecology lab at the University of Chile for species and sex identification using molecular tools. DNA was extracted following a modified salt-extraction protocol [29]. 

### 2.2. Species Identification

The control region of the mitochondrial DNA was chosen for its appropriate mutation rate for species identification [30] and extensive use in cetacean genetic studies [31]. The primers described by [32] were used: M13 Dlp1.5 5′-TGTAAAACGACAGCCAGTTCACCCAAAGCTGRA RTTCTA-3′ (forward) and 8G 5′-GGAGTACTATGTCCTGTAACCA-3′ (reverse). A second set of primers targeting the same fragment was designed by our team for porpoises (Phocoenidae), as amplification was unsuccessful with the previous pair of primers. These new primers were 5’-ATTCAAATCTCGCCGCCAACACCCAAAGCTGGAATTCTT-3’ (forward) and 5’AGAGTAGTATGTCCTGTAACCA-3’ (reverse).

Total reaction volume for each PCR reaction was of 25.6 µL: 12.7 µL of water, 5 µL of 10X Buffer (Invitrogen), 2 µL of 50 mM MgCl_2_ (Invitrogen), 2 µL of 10 pM dNTPs (Invitrogen), 1 µL of 10 pM of each primer (2 µL total), 0.5 µL of Taq polymerase (Invitrogen), and 70–150 ng of DNA. For all amplifications, a Thermo Hybaid PxE 0.5 thermocycler was used with the following cycle profile: preliminary denaturation of 2 min at 94 °C; followed by 30 cycles of denaturation for 30 s at 94 °C, annealing for 40 s at 56 °C, and polymerase extension for 40 s at 72 °C; and a final polymerase extension for 10 min at 72 °C and an infinite hold temperature of 4 °C. Each PCR run included positive and negative controls. Fragments were run in a 1% agarose gel, each well containing 3 µL of PCR product mixed with an equal volume of loading dye with 0.3% Gel Red and visualized in a transilluminator (Maestrogen SMU-01). PCR amplicons were sent to Macrogen Inc., Seoul, South Korea, for purification and sequencing with a 3730XL DNA Analyzer (Applied Biosystems). All obtained sequences were manually aligned in ProSeq 3.5 [33]. Species-specific identification for each sample was done using two platforms of comparative analysis of sequences: BLAST (Basic Local Alignment Search Tool, http://blast.ncbi.nlm.nih.gov/Blast.cgi, accessed on 9 April 2023), and DNA Surveillance (http://www.dna-surveillance.auckland.ac.nz, accessed on 9 April 2023) [31]. The former identifies similar regions between sequences, comparing nucleotides with the database and estimating the degree of similarity. The latter is a virtual service for the identification of species via phylogenetic methods, where the uploaded sequence is aligned against validated reference DNA sequences and a distance-based tree is built, allowing the inference of the identity of the sample through its position in the tree. 

### 2.3. Sex Identification

Sex was identified by amplifying the chromosome fragments ZFX and ZFY. The primers used for the X chromosome were P1-5EZ 5’-ATAATCACATGGAGAGCCACAAGCT-3’ and P2-3EZ 5’-GCACTTCTTTGGTATCTGAGAAAGT-3’, and the primers used for the Y chromosome were Y53-3D 5’-ATTTTAGCCTTCCGACGAGGTCGATA-3’ and Y53-3C 5’-CCCATGAACGCATTCAATGTGTGG-3’ [34,35]. PCR reactions were done in a total volume of 21 μL: 7.16 μL of water, 4 μL of 10X Buffer (Invitrogen), 1.6 μL of 50 mM MgCl_2_ (Invitrogen), 2 μL of 10 pM dNTPs (Invitrogen), 1 μL of 10 pM of each primer (4 μL total), 0.24 μL of Taq polymerase (Invitrogen), and 2 μL of DNA at 50–200 ng/μL. The PCR profile was as follows: a preliminary denaturation stage at 94 °C for 2 min; 35 cycles of denaturation at 94 °C for 45 s, annealing at 60 °C for 45 s, and polymerase extension at 72 °C for 60 s; and a final polymerase extension stage at 72 °C for 10 min and a final infinite hold temperature of 4 °C. Each PCR run included a positive control for each sex and a negative control. The sex of each individual was visually identified in a 2% agarose gel by two independent researchers. Females present a single band of approximately 450 base pairs (bp) that corresponds to the X chromosome fragment, while males present an additional band of lower molecular weight (approximately 174 bp) that corresponds to the Y chromosome fragment [34,35]. All findings were biannually reported to SERNAPESCA, including a detailed walkthrough of the data analysis process.

## 3. Results

**Figure 2:**
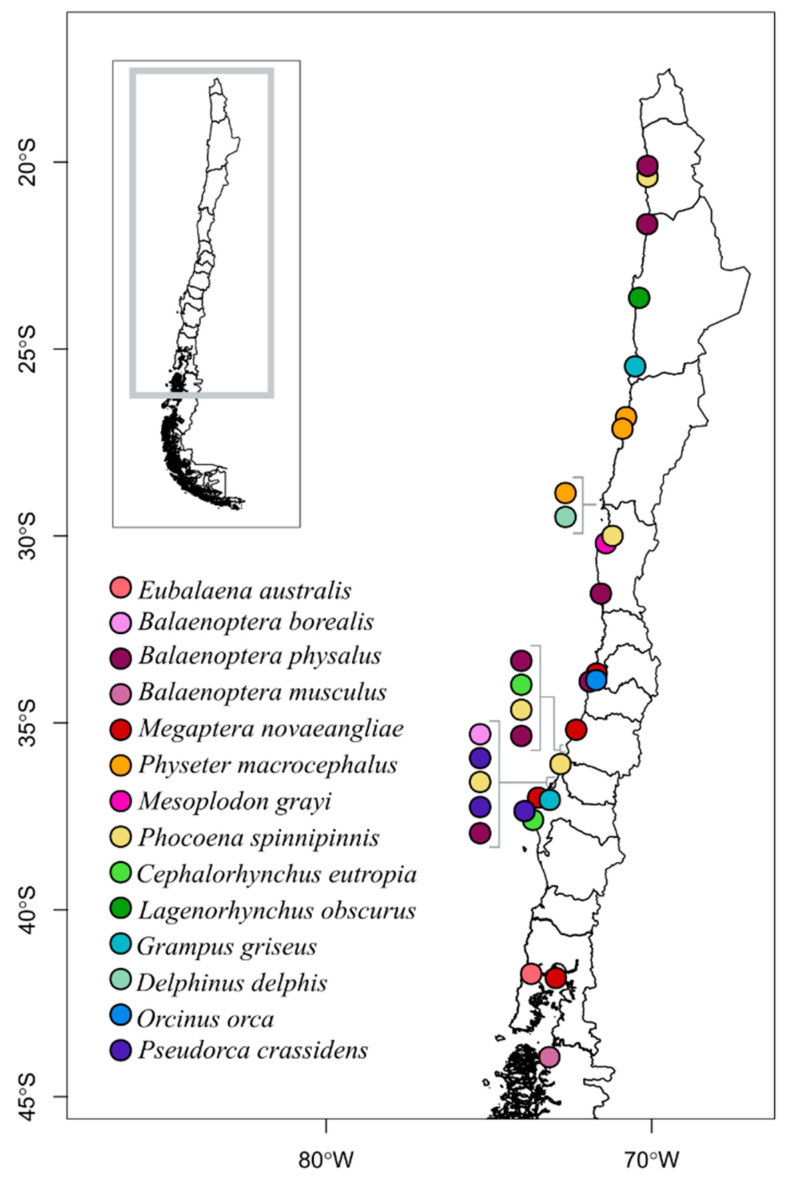
Geographic distribution of the 33 stranded cetacean samples for which PCR amplification was successful and for which a location of origin was provided by SERNAPESCA. The approximate locations of neighboring strandings are indicated with brackets to facilitate the identification of species.

A total of 63 skin samples of cetacean carcasses was analyzed from 2016 to 2021, collected from Iquique, northern Chile (20°10′41.4″ S, 70°8′23.2″ W) to Aysén, southern Chile (41°42′47.5″ S, 73°42′44.5″ W) (Figure 2, Supplementary Material). The control region of 39 (62% of total) samples was successfully amplified, while 24 samples (38%) were not able to be amplified (Table 1). The samples identified to species level corresponded to six families: 17 to Balaenopteridae (5 species), 11 to Delphinidae (7 spp.), five to Phocoenidae (1 sp.), three to Physeteridae (1 sp.), two to Ziphiidae (2 spp.), and one to Balaenidae. The most common species was the fin whale (*n* = 8), followed by Burmeister’s porpoise (*n* = 5) and humpback whale (*n* = 4). Of the 63 samples successfully identified to the species level, 29 (74%) corresponded to matches between field and laboratory identifications (Table 1). Seven samples that could not be identified in the field were successfully identified in the laboratory (18%), corresponding to two *B. borealis*, two *B. physalus*, one *B. musculus*, one *D. delphis*, and one *P. spinipinnis*. Finally, three samples were found to be misidentified in the field (8%), namely two *B. physalus* and a *Mesoplodon grayi* in lab, which were respectively identified in the field as two *B. borealis* and *M. hectori* (Table 1).

The sex of 58 samples was successfully identified (92%). Among these, 20 were corroborated between the sex evaluation done in the field and the laboratory (11 males and 9 females), 34 corresponded to successful assessments for cases of unidentified sex in the field (18 males and 16 females), and four (2 males and 2 females) corresponded to corrections to the sex identification done at the site of the stranding. This information is available in Table 1 for the samples for which species was identified and in the supplementary material for the complete set of samples identified.

## 4. Discussion

The results presented in this study confirm that additional information obtained using molecular tools, for the data collected in the field, in this case species and sex identification, represents a clear source of improvement for the final data set. We showed that laboratory correction of the misidentified individuals as well as the species identification of unknown samples (26%) are crucial, especially for endangered and scarcely studied species. For the samples that were successfully amplified, we provide sex identification for 59% of them (i.e., samples with previously unidentified sex), we confirmed the sex identified in the field for 34% of the samples, and corrected the sex identified in the field in 7% of cases. Therefore, this approach is beneficial for the monitoring of strandings in Chile. 

### 4.1. Species Identification

As mentioned before, the samples identified to species level corresponded to six families (Balaenopteridae, Delphinidae, Phocoenidae, Physeteridae, Ziphiidae, and Balaenidae). Six species of conservation interest were identified using the molecular identification protocol: fin whale, blue whale, sei whale, Burmeister’s porpoise, Chilean dolphin, and southern right whale. Two fin whales were identified as sei whales in the field, probably due to their morphological similarity, which can complicate the recognition of diagnostic characteristics on site. This species is currently catalogued as Vulnerable worldwide [36] and Critically Endangered in national waters [37]. This species was historically affected by intense whaling, and is currently widely affected by vessel collisions worldwide [36] and in Chilean waters [38,39]. In fact, this was the most frequent species in the strandings included in this study, and its correct identification is relevant to correctly estimate their mortality in the national record.

Another relevant case of species misidentification was the sample identified in the field as Hector’s beaked whale that corresponded to a Gray’s beaked whale. Even though this rarely observed species is catalogued as Least Concern [40], it is seldomly identified alive at sea. They appear to be common in some parts of their distribution such as New Zealand, southern Australia, South Africa, Argentina, Peru, and Chile [40]. In Chile, it is a less frequently recorded species in stranding events [9]. 

Additionally, the molecular identification of *Cephalorhynchus eutropia* and *Phocoena spinipinnis* is also noteworthy. These two species are frequently mistaken throughout their shared distribution area due to similar morphological features such as small size, lack of a prominent rostrum and low dorsal fin. Both species have coastal distributions and are affected by interactions with fisheries in these areas [41,42,43]. For the Chilean dolphin (*C. eutropia*), the only cetacean species endemic to Chile, two population units have been identified along its distribution (North and South populations, [44]). It is catalogued as Near Threatened in [45], and as Vulnerable (North population) and Near Threatened (South population) by the Species classification regulation of Chile (Reglamento de Clasificación de Especies, RCE) [37]. Its elusive and unpredictable behavior makes it difficult to obtain skin biopsies for genetic analyses, so samples from strandings are valuable to increase our knowledge of both populations. 

The Burmeister’s porpoise (*P. spinipinnis*) is endemic to the Pacific and Atlantic coastal temperate waters of southern and central South America [46]. It is currently classified as Near Threatened [47] based on its limited range, low densities, and high mortality in some fisheries [48]. Individuals of this species are frequently found stranded along the Chilean coast [9], often with signs of fisheries interaction, such as scars caused by fishing nets. Similarly to *C. eutropia*, obtaining biopsy samples is challenging due to its erratic or inconspicuous behavior and small group size [49,50], therefore strandings represent the best source of information. The molecular identification of this species was not as straightforward as for others because the first set of primers we used did not amplify the targeted fragment. Complications during species-level identification using molecular methods are not uncommon, as they have also been reported when working with other cetacean family such as Delphininae [23,51]. To tackle this, additional internal primers were designed and successfully used. Future samples of this species obtained through this collaboration will be processed using these additional set of primers, optimizing our workflow.

The successful molecular identification of a southern right whale (*Eubalaena australis*) is also remarkable. No more than 50 mature individuals are estimated to compose the subpopulation of the Eastern South Pacific (Chile-Peru stock) [52,53], which is cataloged as critically endangered by the IUCN. Although other southern right whale populations have gradually recovered, the Eastern South Pacific subpopulation has shown no signs of recovery. The only genetic study undertaken using a sample of this subpopulation (replicate of the skin sample reported here) revealed that the mtDNA control region haplotype was previously observed in the Indo-Pacific, while microsatellites appeared admixed between the Indo-Pacific and South Atlantic [54]. Thus, more samples are necessary to evaluate its connectivity with other populations worldwide and to identify its genetic population identity.

Finally, both *Delphinus* species (*D. delphis* and *D. capensis*) are recorded in Chilean waters, but *D. capensis* has much fewer records which are restricted to the northern part of the country [55]. Because of the difficulty in distinguishing these two species based on morphology, the molecular corroboration of the species becomes pertinent to update their presence and distribution along this coast. In this study, probably due to the bad conditions of the tissue samples and/or required laboratory protocol optimization, the molecular identification was successful for only one of the seven samples collected and is a target for future improvement.

### 4.2. Sex Identification

The identification of sex of the individuals using this molecular technique provided even more new information with a successful sex identification in 58 samples (supplementary material). Of these, the majority (*n* = 34) were identifications of unknown sex, 20 cases were confirmation of the sex identification in the field, and the field identification of 4 samples was corrected. As 66% of the 58 samples for which the sex was successfully assessed corresponded to new information, this confirms the difficulty of visual sex assessments in the field and that molecular tools can complement this type of information obtained from strandings. 

Because sexual dimorphism is often reduced to the genitalia, which are internal and only ventrally visible, the identification of sex in cetaceans using morphology can be difficult as they can remain concealed during visual inspections. An example of this is that most field records used for this study lacked information about the sex of the stranded animals [56]. This information can contribute to detecting sex-specific threats in stranded species and can help improve estimations of sex ratios at the population level. This can be also important to identify trends, describe social dynamics and reproductive strategies [57], and assess vulnerability in population-level studies. Thus, including information on the sex of individuals in stranding events is necessary, once again highlighting the use of molecular tools to complement the morphological identification.

### 4.3. Multidisciplinary Approach and Inter-Institutional Collaboration

The systematic collection of stranding data and samples can contribute to the development of other studies in genetics and other fields, further improving our understanding of the biology and ecology of cetaceans. For example, genetic data of some of the samples obtained through this collaboration have been included in two Master’s theses focused on long-finned pilot whales and sei whales [58,59] and two peer-reviewed publications [13,26]. Stranding samples have also contributed in other study areas, for example, stable isotope analyses [60] and toxicological analyses of trace elements and harmful algal blooms [25,61]. In turn, the long-term application of these protocols and the studies that arise from this can produce data that inform and improve future tasks in the management and conservation of cetaceans in Chile.

In addition to Chile, species identification using molecular tools has been applied systematically elsewhere, which has been not only important to obtain more complete records, but also to support conservation and management measures. Although some institutions are large enough to conduct both large-scale sampling programs and molecular analysis of the samples, such as the National Oceanic and Atmospheric Association (NOAA) of the United States, https://www.fisheries.noaa.gov/west-coast/science-data/swfsc-stranding-collections (accessed on 9 April 2023), many sources agree that collaborations between institutions improve the quality of a stranding database, promote scientific advancement and aid the installment of effective conservation and management measures [5,62,63]. The New Zealand Cetacean Tissue Archive, https://mmeg.wordpress.fos.auckland.ac.nz (accessed on 9 April 2023), is an example of a collaborative program between institutions, which was established in 1991. It now holds one of the largest tissue collections of stranded cetaceans and, among other goals, has advanced the study and management of rare cetacean species [5]. Also, a collaboration between French institutions, including the French Stranding Network, have shown the usefulness of molecular approaches in the monitoring of marine mammal diversity [23]. In Brazil, a collaborative stranding network has collected numerous samples, and a high concordance between morphological and molecular identification methods was found (93%) after integrating the information. Some misidentifications were also corrected and the first sequences of two rare species available in online genetic databases were produced, highlighting the benefit of this multidisciplinary approach [51]. 

Following this, the newly implemented protocol in Chile improved the species and sex information collected in the field, showing the benefits of including molecular analysis. It also sets the beginning of a new data collection strategy that will help local authorities in their important management tasks. The tasks carried out by government agencies such as SERNAPESCA are complex, as maintaining a stranding network requires trained personnel working coordinately over an extensive coast. These efforts could also benefit greatly from including other actors in the sampling process, such as scientific observers in industrial fisheries or trained local and authorized fishermen that know the area and, in many cases, can have faster response times. Despite the sampling rate of 43% of all stranded animals that are reported, there is a lack of samples from localities of difficult access, thus future improvements should include capacity building to improve this.

A better understanding of the diversity of species involved in strandings, their stranding frequency, and sex ratios is essential for the establishment of effective conservation and management decisions, in this case in Chilean waters. Similarly, the correct identification of endangered species can aid in the implementation of targeted conservation measures.

## 5. Conclusions

The use of molecular tools to identify the species and sex of stranded cetaceans and as a complementary method in the monitoring of strandings in Chile was supported in this study. Using tissue samples collected in the field, species and sex were corroborated, misidentifications were detected and corrected, and unidentified samples were identified. Six species of high conservation interest were detected among the analyzed samples. These results were obtained through a collaboration between the Chilean National Fisheries Service and the Molecular Ecology laboratory at the Universidad de Chile, an action that improved the quality of the cetacean stranding record. Considering that cetacean strandings are an important source of information to understand their ecology and to support management and conservation efforts, we suggest this collaboration to be maintained and broadened to include other disciplines. For example, veterinary studies could investigate causes of death via necropsies in a systematic way (currently it is performed opportunistically), contributing to the identification of threats. Finally, improvements to the laboratory protocols will be included to further elevate the quality of the collaboration. These factors would culminate in more robust conservation strategies, tailored to the needs and context of each country. 

## Figures and Tables

**Table 1:** Summary table of the samples for which species identification was successful in the laboratory. Species and sex results from field and laboratory are compared. Samples are grouped by family, indicating the number of samples in parentheses. Species names are based on laboratory results. Match: corroboration of species identification on the field using molecular tools in the laboratory; New ID: sample came unidentified from the field and was successfully identified to the species level in the laboratory; Mis ID: sample came with an incorrect species identification from the field and was corrected in the laboratory; Indet: assessment in the laboratory was not possible. Full table available as Supplementary Material.

Family	Species Identification					Sex Identification			
	Species (molecular ID)	*n*°	Match	New ID	Mis ID	Match	New ID	Mis ID	Indet
Balaenidae (*n* = 1)	*Eubalaena australis*	1	1				1M		
Balaenopteridae (*n* = 17)	*Balaenoptera physalus*	8	4	2	2	1F, 4M	1F, 1M	1M	
	*Balaenoptera bonaerensis*	1	1				1M		
	*Balaenoptera borealis*	3	1	2			1M, 1F		1
	*Balaenoptera musculus*	1		1					1
	*Megaptera novaeangliae*	4	4			1M	1F, 1M	1M	
Delphinidae (*n* = 11)	*Cephalorhynchus eutropia*	2	2				1F	1F	
	*Delphinus delphis*	1		1		1F			
	*Grampus griseus*	2	2			1M	1F		
	*Lagenorhynchus australis*	1	1				1M		
	*Lagenorhynchus obscurus*	1	1			1M			
	*Orcinus orca*	1	1			1M			
	*Pseudorca crassidens*	3	3			2F	1M		
Phocoenidae (*n* = 5)	*Phocoena spinipinnis*	5	4	1		1F	2F, 1M		1
Physeteridae (*n* = 3)	*Physeter macrocephalus*	3	3			1F		1F	1
Ziphiidae (*n* = 2)	*Mesoplodon grayi*	1			1		1F		
	*Mesoplodon layardii*	1	1				1M		
Total		39	29	7	3	14	17	4	4

## Data Availability

The data presented in this study are openly available in GenBank, under the accession numbers OQ924479 to OQ924517.

## Supplementary Materials

The following supporting information can be download at: https://www.mdpi.com/article/10.3390/biology12050748/s1, Table S1: Detailed table of the samples that were analyzed for this study, showing the species and identification on the field (“Field ID” and “Field sex ID”); species and sex identification using molecular tools in the laboratory (“Lab Species ID” and “Lab Sex ID”); if the laboratory species and sex identifications matched, corrected or identified unidentified samples (Spp. ID and Sex ID); the IUCN Red List (“Red List”) and Species classification regulation of Chile (“RCE”) conservation statuses; the locations (“Location”) and dates (“Date”) of the strandings; and the GenBank accession numbers.
